# Health-related quality of life of dialysis patients during early COVID-19 lockdowns: a snapshot from a public hospital in Malaysia

**DOI:** 10.3389/fmed.2023.992870

**Published:** 2023-05-25

**Authors:** Ganesh Sritheran Paneerselvam, Khang Wen Goh, Yaman Walid Kassab, Muhammad Junaid Farrukh, Bey Hing Goh, Pei Lin Lua, Andi Hermansyah, Long Chiau Ming

**Affiliations:** ^1^School of Pharmacy, Taylor's University, Subang Jaya, Selangor, Malaysia; ^2^Faculty of Data Science and Information Technology, INTI International University, Nilai, Malaysia; ^3^Faculty of Pharmacy, Syrian Private University, Damascus, Syria; ^4^Faculty of Pharmaceutical Sciences, UCSI University, Cheras, Kuala Lumpur, Malaysia; ^5^Biofunctional Molecule Exploratory Research Group, School of Pharmacy, Monash University Malaysia, Sunway City, Malaysia; ^6^College of Pharmaceutical Sciences, Zhejiang University, Hangzhou, Zhejiang, China; ^7^Faculty of Pharmacy, Universiti Sultan Zainal Abidin, Besut, Terengganu, Malaysia; ^8^Department of Pharmacy Practice, Faculty of Pharmacy, Universitas Airlangga, Surabaya, Indonesia; ^9^Pengiran Anak Puteri Rashidah Sa'adatul Bolkiah Institute of Health Sciences, Universiti Brunei Darussalam, Gadong, Brunei; ^10^School of Medical and Life Sciences, Sunway University, Sunway City, Malaysia

**Keywords:** hemodialysis, kidney disease, KDQOL-36, quality of life, peritoneal dialysis

## Abstract

**Background:**

End-stage renal disease is the last stage of chronic kidney disease and can affect the quality of life (QOL) of dialysis patients. The aim of this study was to assess the quality of life and examine its determinants.

**Methods:**

A cross-sectional survey involving patients on dialysis in a tertiary hospital was conducted from July 2020 to September 2020. Demographic data were collected using a predesigned questionnaire. QOL was measured using the 36-item KDQOL questionnaire, and statistical analysis was carried out using SPSS version 25.

**Results:**

Of the 108 patients, 59 were men and 49 were women, and the mean age was 48.15 ± 15.4 years. The results showed that there was no significant difference in the mean score of all components of health-related quality of life in different types of dialysis. The demographic data, which included age, gender, ethnicity, marital status, education level, occupation, and monthly income, did not significantly affect the QOL of dialysis patients. Patients with a dialysis duration of more than 5 years had a better QOL compared to other groups. Laboratory parameters such as low albumin and low hemoglobin showed a significant correlation with the health-related quality of life of dialysis patients.

**Conclusion:**

The quality of life among patients on dialysis was impaired, especially in terms of burden of the kidney disease. Hypoalbuminemia and anemia were the two factors that influenced QOL.

## Introduction

End-stage renal disease (ESRD) is the last stage of chronic kidney disease (CKD), and its incidence has doubled over the past decade (1). Malaysia has seen a sharp increase in ESRD due to diabetes in the last two decades; in fact, diabetes is the leading cause of ESRD in the country (2). There are three main treatment options for ESRD patients: hemodialysis (HD), peritoneal dialysis (PD), and kidney transplantation (3). The factors that need to be considered when choosing between dialysis and transplantation are age, related health problems, the availability of a donor, and individual preference. Although transplantation is often the preferred treatment, dialysis plays an important role as it is needed for persons who are lying and waiting for transplantation, and also for patients who are not transplantation candidates (4).

The improvement in the survival of patients with ESRD can be seen due to advances in dialysis treatment (5). However, despite all the advances, the mortality rates of patients due to ESRD remain high (6). Inevitably, most studies show an impaired quality of life in dialysis patients regarding the items that reflect physical and mental health (7). Health-related quality of life (HRQOL) refers to the physical, psychological, and social functioning of a person. It is a significant marker of how the patients are coping with their disease (8).

Chronic dialysis has a big impact on patients' HRQOL, including decreased physical functioning and social interaction, increased risk of depression, muscle weakness, restless legs, and post-dialysis fatigue (9). Assessment of QOL is a predictive indicator of the outcome of the disease, as well as a valuable research tool in assessing the effectiveness of the therapeutic intervention, patients' survival, and hospitalizations (10). End-stage renal failure has a highly negative impact on patients' QOL due to the accompanied impairment and the imposed limitations in almost all domains of their daily lives (11). This disease causes serious complications that have a negative impact on patients' lives and puts their physical, mental, and emotional health at risk. In addition to aging, longer dialysis duration, and physiological stressors, financial constraints impair their QOL (12–14). Dialysis also causes a loss of income and has a bad effect on marital status and family life. Finances may be affected by the high cost of weekly treatments for dialysis and occasional admission to the emergency department due to complications.

Hence, to improve QOL, patients receiving chronic dialysis should receive holistic care, taking into account somatic, mental, and social aspects, which can prolong life and decrease their mortality (15). Better quality of life is significantly linked to social support from spouses, family, friends, colleagues, and the community (16). Most importantly, education and counseling by clinical pharmacists lead to a clinically and statistically significant improvement in the QOL of hemodialysis patients (17).

Generic and disease-specific instruments have been widely used to assess HRQOL in ESRD patients (18). One of the generic instruments is the 36-item short-form health survey (SF-36), which originates from the Medical Outcome Study. SF-36 has been extensively used and validated to assess the HRQOL in general populations (6, 19). However, it is quite lengthy, especially for patients who get tired quickly and have ESRD (20). A simple version of the SF-36 questionnaire has been introduced which is the SF-12, which has the advantage of only having one-third of the items of SF-36 (20). The Kidney Disease Quality of Life (KDQOL) form is the disease-specific HRQOL instrument. The KDQOL long form is the first version of the KDQOL, comprising 134 questions that span 11 kidney disease-targeted scales. This form has a low level of responsiveness because it is too long. Thus, the Kidney Disease Quality of Life short form (KDQOL-SF) was introduced to healthcare personnel, which consists of SF-36 and 43 kidney disease-specific items (20). The KDQOL-36 questionnaire, which is a shorter version of KDQOL-SF, has also been developed. KDQOL-36 is preferred due to the minimal burden on patients and staff. It consists of the SF-12, which measures mental and physical functioning, a symptoms and problems subscale, a burden of kidney disease subscale, and the effects of kidney disease on daily life subscale. The disease-specific instrument is one of the best and most regularly used tools to evaluate the quality of life (21).

During the COVID-19 pandemic, the Malaysian government put in place several policies to stop the spread of the deadly virus. The number of visits to health service centers in hospitals, including hemodialysis polyclinics, was limited. Moreover, the shortage of manpower due to the transfer of some staff from dialysis units to other wards and the requirement for social distancing resulted in some patients being stationed in some private dialysis units. Furthermore, as hemodialysis patients are at high risk of COVID-19, they were required to perform swab tests at least two times a week, which put a financial burden on them. Continuing to receive treatment, strictly adhering to health protocols, and financing COVID-19 swabs, coupled with restrictions on food, drink, and physical activity, naturally created further burdens on hemodialysis patients, which posed an indirect risk of reducing their quality of life. Therefore, in this study, we aimed to assess the health-related quality of life of renal patients undergoing regular dialysis during the COVID-19 pandemic and also examine its determinants.

## Materials and methods

### Study design and population

The study was conducted as a cross-sectional study, which involved ESRD patients on dialysis at a dialysis unit in a city hospital in Malaysia.

### Sample size

A sample size of 113 was calculated via Raosoft software using 5% as a margin of error and 95% as a confidence interval. At least 120 patients were approached to fill out the responses, out of which 117 agreed to participate. A total of nine questionnaires were excluded due to incomplete responses. Hence, a total of 108 patients were recruited for the study.

### Sampling technique

A convenience sampling technique was employed to select the study participants. Patients receiving hemodialysis were recruited after screening according to the inclusion and exclusion criteria. Patients who had completed at least 3 months of HD or PD were aged 18 years and above and of either gender were included in the study. Those with impaired cognitive function or who were transferred to another center were excluded.

### Data collection procedure

Permission was obtained to conduct this study in a dialysis unit, with ethical approval from the Medical Research and Ethics Committee. The eligible patients were approached and a brief explanation about the purpose of the study was given. They were also told about their rights to participate in and withdraw from the study. Data collection only started after patients signed the informed consent form to agree to the research. Patients were given a self-administered questionnaire to assess their QOL using KDQOL for completion. Each patient was given about 15–30 min to complete the questionnaire at their own pace. This study did not interfere with nor intervene in patients' disease management.

### Study instruments

The QOL index was measured using 36 items in the Kidney Disease Quality of Life-36 (KDQOL-36) questionnaire. KDQOL-36 is a disease-specific instrument that has been widely used to assess the HRQOL of ESRD patients. Therefore, this tool can be considered reliable and have good reproducibility. Moreover, the tool is available in a wide variety of languages and is easily accessible to researchers. The KDQOL-36 contains a subset of the KDQOL-SF items: the SF-12 items and 24 items to obtain three kidney disease-specific scales, which are the burden of kidney disease (four items), the effects of kidney disease on daily life (eight items), and symptoms or problems (12 items). The three disease-specific subscales are summed into the kidney disease component summary (KDCS) score. The SF-12 is developed using a subset of the SF-36 items. It generates two summary scores, which are the physical component summary (PCS) and the mental component summary (MCS). All these scores range from 0 to 100, with higher scores reflecting better health. The English and Malay versions of the KDQOL-36 questionnaire were used for this study. The patient's clinical characteristics, laboratory results, and demographic data were obtained through the e-His live system that was available in the hospital. These data were recorded in the data collection form.

### Statistical analysis

Data were analyzed using version 25.0 of SPSS statistical software (SPSS Inc., Chicago, IL, USA). *P*-values <0.05 were considered to indicate statistical significance. The demographic data were analyzed by using a descriptive statistic test. An independent sample *t*-test and ANOVA test were used to compare the mean score in the data with normal distribution. In addition, a Pearson correlation test was used to identify the association between the laboratory parameters and components of HRQOL.

## Results

A total of 108 dialysis patients were included in this study. Their socio-demographic and disease-specific characteristics are shown in Table 1, while the laboratory parameters are tabulated in Table 2. The patients' mean (SD) age was 48.15 ± 15.48 years, with 54.6% men, 78.7% Malay, 71.3% married, and 66.7% with a lower level of education. A high proportion (45.4%) of the patients were unemployed, or with a monthly income of less than RM500 (57.4%). There were about 58.3% PD patients and 41.7% HD patients with a dialysis vintage of 2–5 years. From Table 1, the results demonstrated that there was no significant difference in the mean score of HRQOL components for all the demographic data except for the duration of dialysis. The results demonstrated that the mean difference in the MCS between the duration of dialysis of <2 years, and more than 5 years was statistically significant with a *p*-value of 0.029, which showed that patients with a duration of dialysis of more than 5 years have a better QOL mentally. The types of dialysis and the number of comorbidities did not have any significant impact on the HRQOL of the patients.

**Table 1:** HRQOL scores by general characteristics.

	***N* (%)**	**Physical component summary (PCS)**	**Mental component summary (MCS)**	**Burden of kidney disease**	**Symptoms of kidney disease**	**Effect of kidney disease**
Age	48.15 ± 15.48^a^	−0.149	0.055	−0.138	−0.020	−0.078
**Gender**						
Male	59 (54.6)	54.58 ± 27.37	67.49 ± 22.38	45.55 ± 25.95	74.72 ± 14.58	68.70 ± 19.85
Female	49 (45.4)	53.32 ± 27.66	69.18 ± 19.96	51.66 ± 29.39	74.45 ± 16.87	71.88 ± 21.88
**Ethnicity**						
Malay	85 (78.7)	52.34 ± 27.33	67.28 ± 21.51	49.41 ± 26.98	74.24 ± 15.80	70.59 ± 20.91
Others	23 (21.3)	60.14 ± 27.32	71.85 ± 20.24	44.29 ± 30.09	75.91 ± 15.01	68.48 ± 20.53
**Marital status**						
Single	17 (15.7)	63.19 ± 23.42	70.69 ± 19.61	53.68 ± 29.82	77.08 ± 13.66	75.55 ± 19.93
Married	77 (71.3)	54.11 ± 27.22	68.47 ± 21.53	48.62 ± 27.91	74.08 ± 15.81	68.55 ± 20.71
Others	14 (13.0)	42.26 ± 30.13	64.11 ± 22.39	40.18 ± 22.70	74.40 ± 17.28	72.32 ± 22.16
**Educational level**						
Low	72 (66.7)	52.14 ± 27.83	67.74 ± 21.48	46.53 ± 28.88	74.25 ± 15.48	68.66 ± 21.92
High	36 (33.3)	57.73 ± 26.45	69.28 ± 21.00	51.91 ± 24.86	75.29 ± 15.98	73.09 ± 18.15
**Occupation**						
Employed	38 (35.2)	57.98 ± 27.70	67.11 ± 21.16	53.13 ± 23.19	75.93 ± 16.15	71.79 ± 18.66
Unemployed	49 (45.4)	51.11 ± 26.39	68.62 ± 20.77	47.70 ± 30.88	75.21 ± 15.67	72.70 ± 21.54
Retired	21 (19.4)	53.57 ± 29.50	69.48 ± 23.34	41.07 ± 26.34	70.73 ± 14.41	61.16 ± 21.02
**Monthly financial income**						
RM0–RM499	62 (57.4)	52.96 ± 25.63	67.62 ± 21.11	46.88 ± 30.30	74.80 ± 15.05	70.46 ± 22.82
RM500–RM2000	19 (17.6)	50.39 ± 31.42	65.92 ± 24.43	47.37 ± 23.87	71.27 ± 17.53	65.79 ± 15.81
RM2001–RM5000	27 (25.0)	58.95 ± 28.70	71.36 ± 19.54	52.31 ± 23.78	76.47 ± 15.59	72.45 ± 18.93
**Duration of dialysis**						
<2 years	33 (30.6)	50.25 ± 25.73	60.96 ± 22.44^*^	41.86 ± 30.07	72.16 ± 18.36	69.22 ± 24.11
2–5 years	40 (37.0)	57.08 ± 27.59	69.06 ± 22.26	52.03 ± 28.92	73.59 ± 17.06	71.56 ± 19.47
>5 years	35 (32.4)	54.02 ± 28.96	74.21 ± 16.96^*^	50.18 ± 22.96	78.04 ± 9.70	69.38 ± 19.25
**Types of dialysis**						
HD	45 (41.7)	56.46 ± 26.73	68.04 ± 21.63	46.53 ± 26.23	75.88 ± 12.05	67.71 ± 18.33
PD	63 (58.3)	52.25 ± 27.92	68.41 ± 21.12	49.60 ± 28.68	73.68 ± 17.72	71.88 ± 22.31
**Comorbidities**						
≤ 2	67 (62.0)	57.20 ± 27.59	69.61 ± 21.90	51.59 ± 27.29	75.87 ± 15.72	70.10 ± 20.06
≥3	41 (38.0)	48.78 ± 26.55	66.04 ± 20.16	42.99 ± 27.61	72.51 ± 15.33	70.20 ± 22.10

a Age was expressed as mean ± SD and its association with HRQOL was analyzed by Pearson's correlation coefficient.

* *p* < 0.05 is statistically significant.

**Table 2:** Correlation between laboratory parameters and patients' HRQOL.

	**Physical component summary (PCS)**	**Mental component summary (MCS)**	**Burden of kidney disease**	**Symptoms of kidney disease**	**Effect of kidney disease**
Albumin	0.296^*^	0.022	0.139	0.138	0.070
Calcium	0.081	−0.045	0.059	0.087	0.071
Serum creatinine	−0.008	−0.008	0.114	0.079	−0.005
Hemoglobin	−0.031	−0.101	−0.169	−0.134	−0.244^*^

* Pearson correlation, *p* < 0.05 is statistically significant.

The correlation between laboratory parameters and HRQOL components is tabulated in Table 2. From the table, it can be seen that albumin has a positive correlation with the PCS domain, and it is statistically significant (0.296^*^), indicating that higher albumin levels are associated with improved physical health. Calcium has little correlation with the prevalence of kidney disease because it has only weak positive correlations with all three domains of the BKD section of the questionnaire. For serum, creatinine has no significant correlation with the PCS and MCS domains but has a weak positive correlation with the BKD (0.114) and SKD (0.079) domains. Lower hemoglobin levels are associated with a greater burden of kidney disease, as evidenced by the statistically significant negative correlation of −0.244^*^ between hemoglobin and the burden of kidney disease. Overall, Table 2 suggests that HD patients' quality of life can be affected by albumin and hemoglobin.

## Discussion

According to Table 1, there was a weak positive relationship between age and MCS which indicated that HRQOL increased with age. The results obtained were in accordance with a study that showed that older patients had a higher MCS score compared to younger patients. This might be because the older patients had greater adaptation to the dialysis treatment and lower expectations in comparison to younger patients (18). The younger patients might discern the dialysis treatment as a challenge and loss that could lead to a lower MCS score (22). Even though there was a weak correlation between age and all HRQOL components, this relationship was statistically insignificant.

All the HRQOL components of dialysis patients showed that the gender difference did not have any effect on their quality of life as the mean difference in the HRQOL components between men and women was statistically insignificant. This was similar to a study conducted in India which showed that gender did not cause any significant difference in the dialysis patients' HRQOL (23). Ethnicity did not have any impact on the HRQOL of the patients, which was in agreement with the findings of another study which proved that the five dependent variables of interest (PCS, MCS, burden of kidney disease, symptoms and problems, and effects on kidney disease on daily life) did not significantly affect the independent variable, ethnicity (24). Differences in marital status, educational level, occupation status, and monthly income also did not have any significant impact on the HRQOL of dialysis patients. These results are supported by many studies that demonstrated similar findings (25–27).

From Table 1, there was a significant difference in the mean score of MCS between the duration of dialysis <2 years and more than 5 years. The patients who had a longer dialysis duration had a higher MCS score compared to patients with a duration of <2 years, which indicated that the latter had a lower HRQOL. This was in accordance with a study that reported that significantly high MCS scores were found in patients with a longer dialysis vintage (28). The reason for this might be that the patients had already adjusted, processed, and integrated the psychological demands of the illness as they had undergone the dialysis treatment for a long time, while those in the early stages of dialysis were still trying to adapt to these demands. Such processes of cognitive adaptation were also seen in other patient groups, which helped in improving mental health (18).

The difference in the mode of dialysis did not cause any significant difference in the patient's HRQOL. This might be due to the fact that, regardless of the mode of dialysis that the patients used, they still needed to undergo the treatment for their entire lives. This knowledge itself could badly affect patients' HRQOL, no matter whether they were undergoing hemodialysis or peritoneal dialysis. This result was similar to the finding of another study which found no significant difference in the comparison of HRQOL between HD and PD patients in either mental or physical processes (29).

There was a significant positive correlation between albumin and PCS score. This showed that the HRQOL of dialysis patients in terms of PCS decreased along with the albumin level. This was in accordance with a prospective study, which found a statistically significant positive correlation between albumin and PCS score in dialysis patients (30). The same study also stated that hypoalbuminemia had been shown in most dialysis patients. The significant result in the PCS score was possibly due to muscle weakness and fatigue that occurred in the presence of hypoalbuminemia. This was confirmed by a cross-sectional study that reported a higher level of fatigue experienced by patients who had a low albumin level (24).

Based on the results, the correlation between hemoglobin and the effect of kidney disease was statistically significant. However, it was a weak correlation. This finding was in accordance with a cross-sectional study that proved the effect of kidney disease was significantly correlated with hemoglobin levels (31). A lower level of hemoglobin was the main cause of anemia, which was a common complication of CKD. A decrease in erythropoietin production in the peritubular cells of the kidney was a major factor that could lead to anemia. A progressive reduction in hemoglobin concentration will occur in the presence of renal function impairment. Many symptoms could arise as a result, including lethargy and tiredness, breathlessness upon exertion, and muscle fatigue (32). Some of these symptoms had a significant correlation with the effect of kidney disease.

Hypoalbuminemia and anemia are known to have a negative impact on HD patients' quality of life, and their occurrence during the COVID-19 pandemic had a significant impact on HD patients' lives. During the COVID-19 pandemic, HD patients were at increased risk of severe illness and death if they contracted the virus. In addition, the COVID-19 virus could cause a systemic inflammatory response that could worsen hypoalbuminemia in HD patients, leading to further complications. Similarly, anemia could exacerbate the risk of severe illness and complications in HD patients who contract the virus, as it can weaken the immune system's ability to fight off infections and increase the risk of complications such as acute respiratory distress syndrome. This study highlights that HD patients with hypoalbuminemia and anemia were at an increased risk of severe illness and poor outcomes if they contracted COVID-19. Hence, it was crucial to monitor albumin and hemoglobin levels in HD patients and take appropriate measures to prevent and manage anemia as well as hypoalbuminemia, to reduce the risk of complications during the COVID-19 pandemic.

This study has some limitations that need to be mentioned. It was carried out in one particular HD unit in Malaysia, thus, its findings cannot be generalized to hemodialysis patients' overall quality of life during the COVID-19 pandemic. In addition to that, the data were collected using self-administered questionnaires, which could have resulted in response bias due to changes in the respondents' attention and motivation. Having said that, response bias was minimized by using a validated questionnaire, simple language, and ensuring that the patients felt comfortable before starting to answer the questionnaire. The strength of this study was that the researcher was not a member of staff in the HD unit so he could not influence the recruited patients, since he could only give neutral answers.

This study also highlights that healthcare professionals can better tailor treatments to individual patients, and take measurable steps in monitoring and treating HD patients, leading to improved outcomes and a better quality of life. This can have a positive impact not only on the patients themselves but also on their families and caregivers, as well as on society, by reducing the burden of kidney disease on the healthcare system.

## Conclusion

In this study, age, gender, ethnicity, marital status, education level, occupation status, and monthly income did not show any significant effect on patients' HRQOL, except for the duration of dialysis or dialysis vintage. Patients with a longer dialysis vintage had a better HRQOL compared to those with a shorter duration of dialysis. Hypoalbuminemia and anemia demonstrated a significant correlation with HRQOL. From this study, it can be concluded that the HRQOL of dialysis patients is impaired. It is hoped that this finding will be utilized by nephrologists and other healthcare providers to deliver motivational counseling, in order to improve patients' wellbeing.

## Data availability statement

The raw data supporting the conclusions of this article will be made available by the authors, without undue reservation.

## Ethics statement

The studies involving human participants were reviewed and approved by Medical Research & Ethics Committee. The patients/participants provided their written informed consent to participate in this study.

## Author contributions

Conceptualization, methodology, supervision, and writing—original draft: GSP and AH. Data curation, funding acquisition, and validation: KG and LM. Formal analysis: YK. Investigation: GSP, YK, and BG. Visualization: LM. Writing—review and editing: GSP, YK, BG, MJF, PL, and LM. All authors contributed to the article and approved the submitted version.
